# Influence of Metabolic Dysregulation in the Management of Depressive Disorder—Narrative Review

**DOI:** 10.3390/nu16111665

**Published:** 2024-05-29

**Authors:** Paulina Jakubowska, Marta Balcerczyk-Lis, Milena Fortuna, Aleksandra Janiak, Adrianna Kopaczyńska, Sylwia Skwira, Ewelina Młynarska, Jacek Rysz, Beata Franczyk

**Affiliations:** 1 Department of Nephrocardiology, Medical University of Lodz, ul. Zeromskiego 113, 90-549 Lodz, Poland; 2 Department of Nephrology, Hypertension and Family Medicine, Medical University of Lodz, ul. Zeromskiego 113, 90-549 Lodz, Poland

**Keywords:** depression, obesity, inflammation

## Abstract

Depressive disorders are heterogeneous in nature, and their global reach makes them the cause of suffering for a million individuals worldwide. Standard treatment does not work for one in three people, and side effects can significantly reduce the quality of life. A multidisciplinary approach allows for a broader insight into the nature of the disease, given its complex etiology. One of its elements is the hypothesis of inflammation, which also accompanies obesity-related disease. Obesity and depression interact, causing many researchers to develop new non-pharmacological treatment methods for both diseases. One suggestion is physical exercises that have great potential to be used in clinical practice. They can exert changes on the central nervous system and thus modulate mood. Another is diet, which concentrates on active molecules that also affect the central nervous system (CNS). There is an urgent need to create appropriate criteria and recommendations that systematize existing knowledge and allow it to be used in practice. There is an urgent need to create appropriate criteria and recommendations that systematize existing knowledge and allow it to be used in practice.

## 1. Introduction

Depressive disorders are the most commonly diagnosed category of mental illness, characterized by depressed mood and anhedonia [1,2]. They are a major economic burden on the country and carry the risk of unemployment. The annual cost of treating major depressive disorder (MDD) is $92.7 billion, and just under half of $43.8 billion is spent on treatment-resistant depression (TRD) [3]. 

The first line in outpatient settings is second-generation antidepressants, based on the mechanism of visual inhibition of neurotransmitters, such as selective serotonin reuptake inhibitors (SSRIs) [4]. SSRIs are most often chosen because of their tolerability profile, or more convenient dosage—once a day. Another, but somewhat less commonly used group is tricyclic antidepressants (TCAs), which have a lower price, and greater availability, but are not tolerated so well [5]. There is considerable concern about symptoms caused by anticholinergic effects, which affect compliance. They are usually used for drug-resistant depression [6]. In MDD, it is estimated that 10–60% of patients discontinue treatment [7]. In addition, the effects of the drugs appear after a 6-week expectation, which prolongs the therapeutic process [8]. The efficacy of SSRIs is estimated comparable to placebo [9]. 

The pathomechanism of depression is unclear, but it is possible to distinguish the theory: “the monoamine, the structural and functional brain remodeling”, associated with dysfunction; the “hypothalamic-pituitary-adrenal (HPA) axis, or genetic and epigenetic; and the social psychological aspects”, as well as the increasingly important inflammatory hypothesis” [10]. Depression can also predispose to neurodegenerative diseases such as Alzheimer’s disease [11]. It is associated with somatic diseases such as heart disease, stroke, diabetes mellitus, asthma, cancer, arthritis, and osteoporosis and is bridged by inflammation, which is a risk factor for mood disorders [12,13]. Immune processes and also neurotransmitters are influenced by diet, which can produce similar effects to antidepressants [14]. Nutrition interventions, assuming a balanced diet and also being rich in appropriate active ingredients, are an attractive option among depressed patients struggling additionally with obesity, whose eating habits are usually incorrect. The co-occurrence of the two diseases is dictated by inflammation, the broad endocrine system and genetics, and psychological factors like emotional state, and environment [15]. One promising form of therapy is physical activity, which is relatively less common among obese patients. There is evidence pointing to exercise as a supportive or even potential substitute for standard pharmacology or psychotherapy. Further development in this direction, however, requires determination of the type of exercise, the patient group, and the degree of efficacy compared with traditional methods [16]. From a biological point of view, exercise affects neuroplasticity, inflammation, oxidative stress, and areas associated with depression [17].

## 2. Factors of Mental Health

Many factors have a real influence on our mental health and well-being. They can also be variable and unmodified. Among the variables that can be listed: economic factors, social factors, sleep quality, obesity, etc. Unmodified factors are sex differences, age, race, etc. Considering economic factors, research based on the economic crisis of the last decades indicates that men are more likely than women to develop poor mental health. They have a greater risk of developing depression and committing suicide. Rising unemployment, staff reduction, and other economic stressing factors connected with poor family relationships or no public welfare are claimed to be crucial [18]. However, the proportions regarding gender and depression are reversed, as women are twice as likely to suffer from this condition [19]. The prevalence of this diagnosis in women is caused by a higher percentage of sexual abuse than in men or hormonal disturbances that cause stress responsivity [20]. 

The importance of quality sleep is widely known for both mental and physical well-being. Disorders affecting mood, like depression, often disrupt our body’s natural sleep-wake cycle. Sleep issues might also contribute to a higher risk of depression returning after treatment. The correlation between sleep and depression seems to work both ways, with insomnia potentially worsening depression and depression potentially exacerbating sleep problems. For that reason, nowadays insomnia is treated comprehensively using pharmacotherapy, strategies based on sleep hygiene techniques, and cognitive-behavioral techniques [21]. Social relationships may have a positive and negative effect on well-being and depression. The partners can fulfill or undermine the person’s basic psychological needs, directly affecting the depressed individual’s well-being. The influence should be considered as a bidirectional relationship in which depressed people also contribute to the generation of interpersonal stress. It results in the deterioration of the quality of interpersonal relationships and eventually decreases social support for depression [22].

Obesity and depression are strictly associated, according to epidemiological evidence. They are linked through many mechanisms, including biological pathways, genetics, microbiome, neuroendocrine regulators, HPA axis, and immuno-inflammatory activation. One of the main difficulties in treatment results from a different relationship between depression and obesity: dysregulation in their biological mechanisms, in every patient [23]. Additionally, obese patients are more likely to develop depression through their problems with sleep. 

Obstructive sleep apnea (OSA) is repetitive: closure of the upper airway and oxygen desaturations linked with sleep fragmentation may lead to depression. Increased obesity and the aging of the population contribute to the prevalence of OSA [24]. It affects both females and males. The difference between both genders in OSA decreases with age. According to research, women with and without OSA show no significant differences between groups with anxiety and depression. The presence of OSA is not as important as sex differences in personality characteristics in contributing to the clinical spectrum among women. In the same research, the man with OBS reported hypertension (HTN) more frequently than the man without OSA. Central fat mass (CFM) was the only parameter with a nonsignificant increase in men with OSA [25].

## 3. Pathophysiology

The etiopathogenesis of both depression and obesity involves complex interactions between genetic, environmental, and psychological factors. While depression and obesity are separate health issues, they can mutually exacerbate one another [26]. Both conditions exhibit a polygenic characteristic. 

Some of the main biological pathways linking obesity with depression are inflammation, hypothalamic-pituitary-adrenal axis (HPA) hormonal disturbances, and neuroprogression [27]. 

As obesity is known as a chronic proinflammatory state, the latest research also has proven higher levels of inflammatory cytokines such as C-reactive protein or interleukin 6 (IL-6) in depressed individuals compared to non-depressed adolescents [28]. Depressive manifestations facilitate weight gain, which subsequently triggers an inflammatory response through the release of IL-6 from adipose tissue and the leptin-induced increase in interleukin-6 released by white blood cells [29]. Chronic inflammation also contributes to indoleamine 2,3-dioxygenase (IDO) activation, which is connected to tryptophan (TRY) metabolism, driving the production of its catabolites (TRYCATs). Activated TRYCATs are assumed to be another potential path for the development of mood symptoms in obese patients [30]. Moreover, there has been a notable elevation of pro-inflammatory cytokines in brain areas linked with mood disorders, such as the hippocampus and hypothalamus, in both states [31]. 

Dysregulation of the HPA is another pathophysiological link between depression and obesity. The hyperactivity of this axis has been found in 40–60% of individuals with major depressive disorder (MDD) [32]. Cytokines such as IL-6 stimulate the HPA axis to promote obesity or insulin resistance through a network of mechanisms involving the resistance of glucocorticoid receptors to cortisol [30,33]. This mechanism suppresses inhibitory control of the sympathetic nervous system, leading to inflammation [33]. 

Neuroendocrine substances such as ghrelin and leptin are involved in regulating appetite and energy balance, which are significant in the context of obesity and MDD. Both hormones are altered in obesity, where they contribute to energy imbalance through effects on appetite stimulation, metabolism, and inflammation [33]. In individuals with obesity, leptin resistance and dysregulated ghrelin secretion may contribute to mood disturbances and increase the risk of depression [26,33].

Moreover, MDD can contribute to insulin resistance, which in turn heightens the risk of exacerbating depressive symptoms [32]. Insulin plays a role in modulating reward behaviors, and various brain regions sensitive to insulin are linked to depression development [32]. Insulin resistance leads to obesity and, subsequently, to experiencing victimization, which is one of the components associated with depression [34].

The relationship between depression and obesity might also extend through the hypothalamic-pituitary-thyroid (HPT) system [33]. Free thyroxine hormone 4 (FT4) negatively affects BMI, further resulting in a higher prevalence of depression in obese patients [35]. 

Another method to investigate the shared genetic basis between depression and metabolic disorders such as obesity involves exploring gene-environment interactions. It was found that a variation in the promoter region of the serotonin transporter gene SLC6A4 influences the likelihood of depression among individuals confronted with challenging life events [36]. 

Among the pathways implicated, environmental factors play an essential role in the pathogenesis of both depression and obesity. Certain modifiable lifestyle behaviors, like reduced physical activity and an unhealthy diet, are linked to an increased risk of both obesity and anxiety [37]. In the research, men exhibiting high levels of visceral fat experienced a greater than twofold increase in the risk of depression compared to those with typical visceral fat levels [38]. Moreover, the link with depressive symptoms was consistently more closely associated with abdominal obesity than with general obesity [39]. 

## 4. Gut-Brain Axis

**Figure 1:**
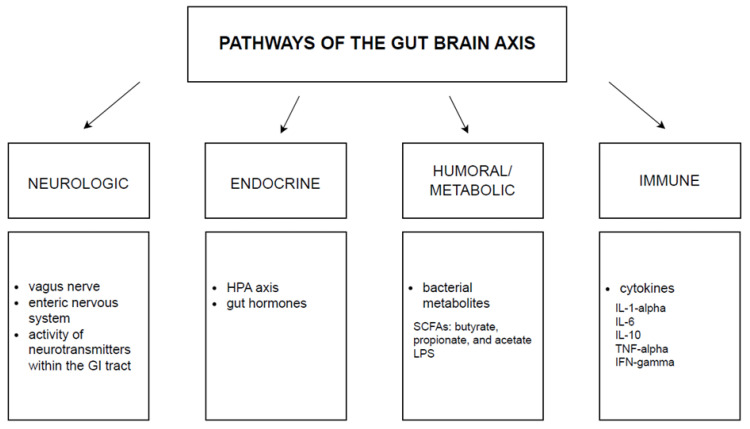
The glut-brain axis. The above pathways mediate bidirectional communication between the brain and the gut microbiota. GI: gastrointestinal tract, HPA: hypothalamic-pituitary-adrenal axis, SCFAs: short-chain fatty acids, LPS: lipopolysaccharide, IL: interleukin, TNF: tumor necrosis factor, IFN: interferon.

In recent years, the meaning of the gut-brain axis has significantly grown. In 1980, when the idea of this axis occurred, it was initially described just as a concept; however, nowadays, with all its components, we may entitle it as a system [40]. It is a bidirectional mechanism connecting the intestinal ecosystem and the central nervous system. This complex binding may influence mood and contribute to disruptions in mental health [41]. Although we can list pathways that form gut-brain communication, there are still many mechanisms to discover. The four major pathways taking part in this system are neurologic, endocrine, humoral/metabolic, and immune as presented in Figure 1 [42].

Gastrointestinal tract microbiota can be classified into the groups of yeasts, archaea, parasites such as helminths, viruses, protozoa, and bacteria—the last ones are the best characterized. Over 90% of the microbiome consists of Proteobacteria, Firmicutes, Actinobacteria, and Bacteroidetes [43]. According to preclinical data, the commensal bacteria may influence the hypothalamic-pituitary-adrenal axis (HPA). Moreover, it can alter key neurotransmitters considered relevant in the development of depression [44].

Among the numerous components of the microbiota-gut-brain axis that should also be mentioned are the metabolites of the gut microbiota, the central nervous system (CNS), the neuroendocrine/neuroimmune systems, the autonomic nervous system (ANS), and the enteric nervous system (ENS) [45]. It is very important to keep a balance between all of them; otherwise, many diseases concerning mental health may also occur [40].

Microbes produce metabolites that enable communication between them and interaction with the gut ANS [43]. The mentioned metabolites are short-chain fatty acids (SCFAs), tryptophan precursors and metabolites, serotonin, catecholamines, GABA, sugars, and vitamins. For good reason, SCFAs are mentioned first. They are extremely important because they mainly affect depressive behavior [45], can penetrate the brain through the blood-brain barrier (BBB), control endocrine cells and their release of gut peptides, and also regulate the synthesis of serotonin in the gut, where approximately 95% of total body serotonin, derived from enterochromaffin cells, is produced [42]. Tryptophan has the potential to be converted into serotonin. However, in inflammatory circumstances, a significant portion of tryptophan is instead converted into kynurenine, which can undergo further transformations, leading to the production of anthranilic acid, kynurenic acid, and quinolinic acid. Among these, kynurenic acid (KYNA) and quinolinic acid (QUIN) exhibit properties that modulate the nervous system [46]. KYNA and QUIN are polar and, therefore, ineffectively cross the BBB and must be created in the brain [47]. However, the research conducted on mice indicates that the gut microbiota can influence BBB integrity. In germ-free mice, there was increased transmittance of the BBB, which means the gut microbiota may be a potential regulator of BBB permeability [48].

There have been many recent studies proving a correlation between the gut microbiota and the pathogenesis of depression. Patients with major depressive disorder (MDD) manifest abnormalities in the CNS, endocrine, metabolic, and immune pathways [49]. Additionally, studies indicate significant differences in the gut microbiota of patients with MDD compared to controls. There are various results regarding dominant operational taxonomic units (OTUs) and decreased levels of other OTUs among people with depression [50,51,52,53]. Interestingly, our microbiome and, as a result, the gut-brain axis may be influenced by factors like the use of antibiotics, age, health status, and diet [51]. It was observed that MDD patients are more likely to consume more carbohydrates compared to the control group [53]. This intake explains already-known metabolic pathways present in depression, e.g., the pentose phosphate pathway, and the starch and sucrose metabolism pathways [52].

Substances that exhibit psychological effects through their interaction with the microbiome may be called psychobiotics, or at least substances that exhibit psychobiotic characteristics. Previously, psychobiotics were considered just as probiotics and prebiotics; now, according to some authors, psychobiotics should also include any other substance influencing the microbiome [54].

Probiotics are living bacteria with a positive impact on our organisms dosed in ‘colony-forming units’ (CFU), contributing to a balanced gut microbiota. However, if an imbalance occurs and some bacterial species dominate, it can lead to a disease. In research where fecal microbiota was transferred from patients with depression to rats with reduced microbiota, the recipient organisms observed anhedonia, anxiety-like behaviors, and modifications in tryptophan metabolism [55].

Thus, the importance of gut microbiota diversity is enormous, and probiotics are believed to contribute to this variety [56]. Probiotics did not contribute to a greater reduction of symptoms in MDD compared to the placebo group, but a significant difference between the two mentioned groups was observed in mild/moderate depression [38].

The definition of International Scientific Association for Probiotics and Prebiotics describes prebiotics as “a substrate that is selectively utilized by host microorganisms, conferring a health benefit”. The main prebiotics found in a typical diet are soluble fiber, fructooligosaccharides, inulin, resistant starch, and galactooligosaccharides. They possess many health benefits, not only exclusively for the gastrointestinal (GI) tract but also for the brain [41]. In studies on mice, it was reported that prebiotics were capable of modifying mice’s actions and chemistry in the brain relevant to depression. Moreover, administered prebiotics reduced levels of stress-induced plasma corticosterone [57]. Prebiotics can also decrease neuroinflammation and BBB permeability [41]. It is claimed that prebiotics in the future may be more potent than probiotics. Further research on the usability of prebiotics in the treatment of neuropsychiatric diseases is necessary, according to some authors [58].

## 5. Role of Diet

A person with a high body-mass index (BMI, the weight in kilograms divided by the square of the height in meters) has a higher risk of mortality [59]. According to the World Health Organization (WHO) guidelines for adults, overweight is identified as having a BMI equal to or greater than 25, while obesity is characterized by a BMI equal to or greater than 30 [60]. Three primary elements contribute to the regulation of body weight: the metabolic processing of nutrients, dietary patterns, and levels of physical activity [61]. There is strong evidence that balanced diets like low-fat, low-carbohydrate, and Mediterranean diets can help with managing metabolic conditions [62]. Efforts to prevent obesity should prioritize the maintenance of weight loss or the management of excessive weight gain [63]. Research indicates that weight reduction is linked to a decrease in depression scores. Sustained weight loss within the range of 5–15% of the initial weight is highly advantageous from a medical standpoint, especially when it endures over time [64]. A meta-analysis revealed a two-way relationship between obesity and depression, suggesting that the presence of one condition increases the risk of developing the other [65]. Atypical depression is characterized by inflammatory and metabolic dysregulation, whereas melancholic depression is marked by hyperactivity of the HPA axis. Metabolic abnormalities noted in atypical depression comprise elevated body mass index (BMI), waist circumference, triglyceride levels, and reduced levels of high-density lipoprotein (HDL) [66]. 

Traditional diets based on grains and vegetables are being replaced by meals that are high in fat and sugar [67]. The recommended range for carbohydrate intake is 45–65% of daily energy; for protein, it is 10–35%; and for fat, it is 20–35%, with a focus on limiting saturated and trans fats [68]. However, in today’s typical diet, fat makes up about 35–40% of the total energy consumed, and in some cases, it can go as high as 60% [69].

Eating foods rich in saturated fats might harm cognition, while foods containing unsaturated fats could benefit cognitive function. Diet could influence cognitive function more significantly in women [70]. The study found that eating lots of fatty or sugary foods can make it harder to remember things that rely on the part of the brain called the hippocampus [71].

People who have both obesity and depression seem to be a unique group among those who are depressed. For them, low-calorie diets (LCD) could be a helpful personalized treatment option. A restricted diet could be especially helpful for those with type 2 depression, where symptoms include increased appetite, weight gain, hypersomnia, and a poor metabolic profile. The improvement in mood for these individuals may be connected to a mix of immune and hormonal factors, as well as social and emotional aspects [72]. A systematic review and meta-analysis of interventional studies show that a low-calorie diet could significantly reduce symptoms of depression in overweight and obese people. Additionally, exercise may be helpful when added to a low-calorie diet for reducing symptoms of depression [73].

A randomized controlled trial examined the long-term effects of an LCD on mental health. Over the course of one year, 115 adults who were obese and had type 2 diabetes were randomly assigned to follow either a calorie-restricted, planned isocaloric low-carbohydrate (LC) diet or a high-carbohydrate, low-fat (HC) diet. Both diet groups also participated in a supervised exercise program, meeting three times a week. This study aimed to investigate how following a low-carb diet with reduced calories compares to a traditional high-carb diet with the same amount of calories over a year-long period. The focus was on mood, specifically depression and anxiety, as well as emotional stress related to diabetes, and overall quality of life in obese adults diagnosed with type 2 diabetes. Among the subjects, total weight loss was 9.5 ± 0.5 kg, and there was not much of a difference in weight loss between the two diets. After reassessment, significant improvements were observed in various aspects, including the Beck Depression Inventory (BDI), Profile of Mood States (POMS), diabetes-specific emotional distress (Problem Areas in Diabetes—PAID), and quality of life dimensions related to diabetes (QoLDiabetes-39), such as diabetes control, anxiety and worry, sexual functioning, and energy and mobility [74].

Dietary patterns influence the development of depression symptoms. The data from 3486 participants (26.2% women, with an average age of 55.6 years) in the Whitehall II prospective cohort were analyzed. A study distinguishes two dietary patterns: whole food (rich in fruit, vegetables, and fish) and processed food (rich in processed meat, chocolates, sweet desserts, fried food, refined cereals, and high-fat dairy products). Five years later, the assessment of self-reported depression was conducted using the Center for Epidemiologic Studies—Depression (CES-D) scale. The results suggested that among middle-aged participants, consuming a diet high in processed foods increased the risk of experiencing CES-D depression five years later. A diet filled with fruits, vegetables, and fish may help protect against depression. On the other hand, consuming a diet high in processed meats, chocolates, sugary desserts, fried foods, refined cereals, and fatty dairy products appears to increase the risk of depression in a large prospective cohort of White, middle-aged British participants. Following healthy eating rules will have advantages for health. Improving diet may help prevent depressive disorders [75].

### 5.1. Mediterranean Diet

**Figure 2:**
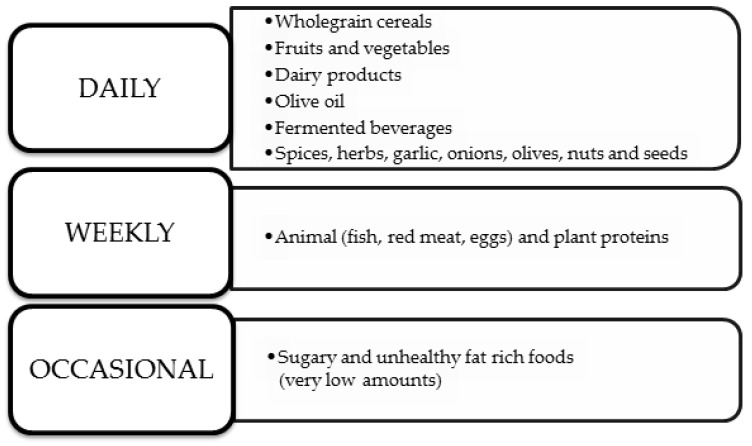
MedDiet’s nutrition rules [78].

Ancel Keys first defined the Mediterranean diet (MedDiet) back in 1960. He presented it as a diet rich in vegetable oils but with a low intake of saturated fats [76]. This dietary model is widely known around the world and is modeled on the diet of people from the Mediterranean region based on whole grains, fruits, vegetables, white meat, and nuts [77,78]. This diet is based not only on choosing the right foods but also on the correct composition of meals, the use of seasonal products, and the correct cooking technique. The frequency of consumption of specific nutrients in the MedDiet is shown in Figure 2 below [78].

It is well known that MedDiet protects against the development of cardiovascular disease, obesity, and diabetes and reduces the risk of developing colon or breast cancer [79]. In addition, due to its anti-inflammatory properties, it benefits mental health [80]. A number of studies have been conducted to see if using the MedDiet alleviates symptoms of depression. In the Healthy Eating for Life with a Mediterranean Diet (HELFIMED) and SMILES studies, clinical improvement was observed in depressed patients in the MedDiet group [81].

HELFIMED is a randomized control trial that evaluated patients reporting symptoms of depression for a minimum of 2 months who were between the ages of 18 and 65 years on the effects of MedDiet on their mental health. Patients in the intervention group changed their diet based on vegetables, fruits, nuts, and grains and minimized their intake of sweets and highly processed foods [82]. In addition, the intervention group received nutrition education and had the opportunity to receive advice from nutritionists and to participate in cooking workshops. Their diet was enriched with fish oil in capsules taken twice a day (450 mg docosahexaenoic acid (DHA) and 100 mg eicosapentaenoic acid (EPA) in 1 dose) [83]. During the study, participants were very adherent to their diets and took into account the advice of the nutritionists involved in the project. Adherence to the diet was observed at a very high level, over 90%. The researchers attribute such a high percentage not only to the workshops conducted or the multi-specialized care but also to the ready-made food packages provided to the subjects [84]. During the joint workshops, participants broke down social barriers, often measuring themselves against their fears. Summarizing the HELFIMED project, it was noted that the subjects found it easier to break down barriers and increased their confidence in interacting with others. It was also shown to have an overall positive impact on participants’ mental health [85]. 

**Figure 3:**
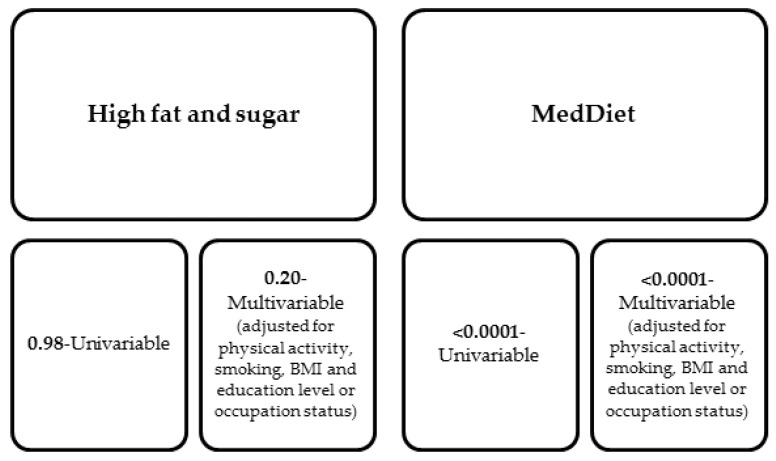
Odds ratio for the association between the occurrence of symptoms on the 10-item depression scale in the group of women using MedDiet and for comparison in the group of women with a high sugar and fat diet [86].

The topic of the effect of diet on the development of depression has also attracted interest in Australia, where a prospective cohort study of more than 40,000 women was conducted (Australian Longitudinal Study on Women’s Health). The study included women aged 45–50, in which the ladies completed questionnaires every 2–4 years, which was supposed to allow researchers to confirm the hypothesis that a Mediterranean diet reduces the incidence of depressive symptoms. In the questionnaires, the women in the study included responses to questions regarding their quality of life, health-seeking behavior, diet, accompanying symptoms (assessed by the 10-point depression scale), or the need for specialized health care. Each group of patients followed a different diet; however, only the MedDiet was shown to be associated with relief of depressive symptoms even despite confounding factors such as smoking and physical activity levels, as shown in Figure 3 below. The study found that the group of women who adhered most strongly to the MedDiet showed 37% less severe depressive symptoms than the group of women who were less strict about dietary recommendations [86].

### 5.2. Anti-Inflammatory Diet

One of the many factors in the development of depression is chronic inflammation. As a result, in recent years, there have been many studies looking for a relationship between taking anti-inflammatory drugs and following a diet that modulates inflammatory factors in the course of depression [87]. In clinical studies, elevated levels of interleukin-6 (IL-6), interleukin-1β (IL-1β), and tumor necrosis factor-alpha (TNF-α), which belong to pro-inflammatory cytokines, have been observed in patients suffering from depression [88].

One of the anti-inflammatory substances taken with food is flavonoids, which belong to polyphenolic compounds [89]. Polyphenols produced by plants serve them for their antibacterial, antifungal, and antiviral effects. In addition, they protect them from ultraviolet radiation or oxidative stress, and, most importantly, from the point of view of their influence on preventing the development of nervous system diseases (including depression), they have anti-inflammatory properties [90]. Flavonoids are mainly found in fruits, vegetables, tea (mainly green tea), nuts, and dark chocolate [89,91]. Among the group of bioactive flavonoids is hesperetin (30,5,7-trihydroxy-40-methoxy-fla-vanone) (Hst), which is mainly found in citrus fruits but can also be found in tomatoes or apples [92]. In experimental studies conducted on mice, the neuroprotective effects of Hst have been noted, and its antidepressant effects have been proven [88]. Researchers have also attempted to test whether Hst can normalize the increased secretion of pro-inflammatory cytokines through the action of lipopolysaccharides (LPS). Indeed, in earlier rodent studies, LPS has been shown to increase oxidative stress, induce neuroinflammation, impair memory, and negatively impact the development of brain inflammation and depression [90]. During the study, significantly higher levels of pro-inflammatory cytokines in the hippocampus and cerebral cortex were examined in the group of mice in which LPS was administered. In contrast, administration of Hst to mice significantly reduced the levels of these cytokines, and interestingly, in a group of mice that maintained both LPS and Htc, it was reported that Htc abolished the negative effects of LPS on both the production of pro-inflammatory cytokines and reactive oxygen species [93]. 

Another well-known substance with anti-inflammatory and antioxidant properties is curcumin, which has been used for centuries for its properties. This world-renowned spice is used supportively in the treatment of arthritis, intestinal inflammation, and diabetes, for example [94,95]. Kulkarni, Dhir, and Akula in 2009, during their studies on rats, noticed and reported an antidepressant effect of curcumin, and Lee and Lee in 2018 demonstrated its anti-anxiety effects. A 2016 meta-analysis by Al-Karawi, Al Mamoori, and Tayyar showed that curcumin alleviated depressive symptoms in patients compared to placebo. Based on studies that take into account the health-promoting and neuroprotective properties of curcumin, it is worth including it in patients with depression as an adjunctive treatment [94].

## 6. Physical Activity 

Physical activity is defined as any bodily movement performed by skeletal muscles that requires energy, for instance, walking, exercises, or even ordinary daily activities [96]. Its participation in daily life can improve mental health by releasing endorphins [97] as well as stimulating angiogenesis, increasing brain perfusion [98], or decreasing the level of cortisol, the stress hormone, which may result in an improvement in patients’ mood [99]. 

Furthermore, physical activity may be a valuable adjunct to conventional treatment methods for various metabolic and psychiatric disorders, including depression. The effects that can be achieved with regular exercise may be comparable to the benefits of antidepressant drugs and psychotherapy [100]. Meta-analyses have demonstrated that for mild to moderate depression, the impact of exercise interventions is comparable to antidepressant medication and psychotherapy (with the most significant effects observed for aerobic exercises of at least moderate intensity, supervised by exercise professionals). However, in cases of severe depression, exercise interventions seem to be a valuable adjunctive therapy [101]. It is widely known that patients using antidepressants showed a reduction in heart rate variability and experienced rises in waist circumference, blood pressure, and triglyceride levels, indicating a potential for an increasing incidence of metabolic syndrome [102].

In a systematic review and meta-analysis, Pearce M. et al. showed an inverse curvilinear relationship between physical activity and episodes of depression. It was specified that the greatest benefits are achieved by those who have moved from a point of lack of physical activity to implementing it, even to a degree below public health recommendations (2.5 h/week of brisk walking) [103]. 

The choice of type of training may be crucial. Patients’ tendencies include individual, instructed training that lasts 30–60 min, with a frequency of several times a week. Walking seems to be the most attractive option, which for obese patients seems to be the optimal option. This is followed by weightlifting, which is preferred slightly more often by men, and yoga, which is more often chosen by women [104].

Exercises dedicated to obese people include those that do not strain the joints, such as swimming or cycling. The latter can be practiced at home. As a result of a virtual reality exercise program with a stationary bicycle, overweight middle-aged women achieved a lower BMI and lower levels of depression. This makes an interesting proposition suitable for people with space or time constraints. An important aspect is the increased level of satisfaction with the exercise, which is a pillar in the further continuation of the therapy [105].

Diabetes often co-occurs with obesity, thus increasing the risk of depression in obese individuals. Here, recreational activity is also mentioned, only this time as a modulator of the diabetes-depression relationship [106].

Systematicity and adherence in the depressed patient population may prove to be a difficulty. A suggestion is HIIT training, which includes short, intensive exercises. This includes short sprint training (sSIT), which causes less muscle fatigue. It turns out that just six sessions of 6–10 min over the course of two weeks can have an impact on MDD symptoms (results measured by HAM-D21). By doing so, increased aerobic capacity, a slight reduction in body fat, and increased levels of spontaneous activity were observed [107].

A study was conducted comparing the effects of antidepressant medication with running therapy on mental and physical health. Out of 141 participants (mean age 38, 2 years; 58, 2% female), 45 received 16-week treatment (escitalopram or sertraline), and 96 underwent running therapy, which consisted of supervised 45-minute outdoor running sessions during 16 weeks. Analyses executed as part of the intention-to-treat showed similar remission rates. However, improvements in physical health indicators were observed in the running therapy group, in terms of changes in body mass, waist circumference, systolic and diastolic blood pressure, heart rate, and heart rate variability [108]. 

Physical training is also considered a significant component of the treatment of obesity in both adults and children due to its numerous benefits [109]. Assessing the physical activity levels of elderly patients might help identify individuals at high risk of developing depressive symptoms [110]. Among adolescents, there is a slight but significantly positive impact of physical activity on mental health. Participation in team sports has shown particularly beneficial effects. Conversely, a sedentary lifestyle is associated with adverse effects on well-being, especially spending more than 2 h per day in front of screens, which correlates with poorer mental health outcomes in children and adolescents [111]. 

Performing aerobic and resistance exercises significantly improves cardiometabolic health [112]. Aerobic exercise training is known to reduce the stiffness of central and peripheral arteries [113,114]. A study was conducted in Spain among 116 overweight or obese children (10.6 ± 1.1 years of age, 53.4% female) aimed to evaluate the effectiveness of physical exercises in the treatment of obesity. They participated in a psycho-education program for 22 weeks, consisting of two family-based education sessions per month, or the same plus supervised exercise. Intense aerobic training three times a week for 90 min resulted in a significant reduction in the percentage of liver fat (as measured by MRI), regardless of the initial value. In addition, body weight reduction and improvements in cardiometabolic indicators also were observed [115]. Early signs of obesity include hypertension and endothelial dysfunction. A 3-month randomized controlled trial was conducted among 44 obese pre-pubertal children (age 8.9 +/− 1.5 years), divided equally into an exercise group and a control group. The first group trained for 60 min three times a week for 3 months, whereas the control group remained inactive. Then both groups trained for the next 3 months. The program did not introduce any dietary interventions. Changes in indicators were assessed after 3 and 6 months. Considerable changes in 24-hour arterial blood pressure monitoring were observed after 3 months. Improvements were noted in indicators such as blood pressure, endothelial function, intima-media thickness, arterial stiffness, and adipose tissue after 6 months [114]. The presented studies are summarized in Table 1. Childhood obesity is also correlated with cardiovascular disease and type 2 diabetes in adulthood [116,117]. The findings indicate that 12-month vigorous or moderate aerobic exercise programs can lead to long-term benefits in preventing diabetes among individuals with central obesity [118]. In a sample of young women, the researchers assessed potential predictors of depression. Cardiorespiratory efficiency, measured in the study via maximal oxygen consumption [VO 2 max] during exercise on a bicycle ergometer, was found to be an independent predictor of Beck scores and, along with the sum of skinfold measurements, correlated moderately with the presence of depression. The researchers acknowledged that measuring the sum of skinfolds is more objective than percent body fat, which is significant in the methodology and comparison of studies [119].

The heterogeneity of the methodology of the resulting studies prevents the development of functional guidelines. A systematic review and meta-analysis showed that physical exercise in an obese population can improve quality of life but does not significantly reduce the severity of depression. The authors acknowledged that the reason was likely due to poor study quality and methodology. They added that it is important to personalize such a set: type, intensity, range of duration, and timing of measuring effects [120].

Based on the presented research, performing aerobic and resistance exercises significantly improves cardiometabolic health [109]. The benefits of physical activity in the prevention and treatment of cardiovascular diseases have been also very well described in adults [121,122]. Based on the analyzed studies, it seems that physical activity should be considered as a significant element of comprehensive treatment for both obesity and psychiatric disorders. The results suggest that regular exercise may bring significant benefits.

## 7. Conclusions

In order to manage both depression and obesity, it is essential to understand their common pathological pathways and seek new ways of dealing with them. A comprehensive and individualized approach is required, and it is important to also take into account the potential advantages of methods such as nutritional interventions [15]. It is also essential to recall the established pharmacological treatment modalities, such as 5-HT3 receptor antagonists, which have an antidepressant-like effect through modulation of the serotonergic system, as a possible therapeutic target for both conditions [18]. Moreover, the newest knowledge about TRYCATs being a possible connection between MDD and obesity is a target to develop novel ways of treatment [22]. An interdisciplinary team of specialists in mental health, nutritionists, and care providers that can offer a wide range of services, including medication, psychotherapy, and lifestyle modifications, is the best way of dealing with both conditions long-term for the patients, remembering about seeking new possible methods [15]. It is important that patients reliably practice movement therapy. The doctor’s job would be to constantly monitor adherence, educate, and monitor a potential exercise diary. Research should be aimed at identifying groups that may benefit most from movement therapy. Observation over an extended period of time of such patients would also be valuable.

Exercise interventions, particularly aerobic exercises supervised by professionals, show promise across different levels of depression. Moreover, in both adults and children, physical training significantly improves cardiometabolic indicators and may prevent conditions like obesity and diabetes. These findings emphasize the vital role of integrating physical activity into healthcare strategies for overall well-being.

## Figures and Tables

**Table 1:** Sampling of studies assessing the impact of physical exercise intervention on psychiatric and metabolic disorders.

Author, Year	*N* (Men/Women), Mean Age	Intervention	Key Results	Methodology
Verhoeven JE, 2023 [108]	58/83, 38.2 years	(1) 16-week treatment: escitalopram or sertraline (2) running therapy ≥ 2 per week	Remission rates at T16 were comparable, but the running group had greater reductions in weight loss, waist circumference, systolic and diastolic blood pressure, and heart rate.	The presence of depressive disorders and/or anxiety disorders was established at T0 and T16 using the CIDI. Severity of depression was measured using the 30-item Inventory of Depressive Symptomatology—Self Report (IDS-SR) and the severity of anxiety was measured with the 21-item Beck Anxiety Inventory (BAI). The physical health outcomes were assessed using outline standardized laboratory methods.
Sofie Holmquist, 2017 [110]	2063/1021, 70 years	Explorative cluster analysis was used to group participants according to functional performance level, using measures of basic mobility skills, gait variability, and grip strength.	One potential high-risk cluster was identified, with an overrepresentation of individuals with GDS scores > 5 (15.1%) also with obese individuals (39.7%) and those with type 2 diabetes (24.7%)	Intercluster differences in depressive symptoms (measured by the Geriatric Depression Scale [GDS]-15), physical activity (PA; measured objectively with the ActiGraph GT3X+)
Labayen I, 2020 [115]	52/62, 10.6 years	First group—family-based education sessions/month Second group—additionally high-intensity aerobic workouts (3 sessions/week, 90 min/session)	Percentage of hepatic fat decreased only in the II group. BMI, abdominal fat, and insulin were reduced in both groups.	Hepatic fat was measured by MRI, abdominal fat was measured by DEXA, and other outcomes were measured by standardized laboratory methods.
Farpour-Lambert NJ, 2009 [114]	n = 44 8.9 years	First group—trained 60 min 3 times/week during 3 months, then 2 times/week during 3 months. Control group—inactive for 3 months, then trained 2 times/week during 3 months.	Decrease in arterial blood pressure after the first 3 months in the I group. After 6 months, endothelial function, intima-media thickness, arterial stiffness, and adipose tissue were lower in the I and the control group.	The pubertal stage was assessed by clinical examination according to the method of Tanner. The DXA was used to do a total body scan. A real-time B-mode ultrasound imager was used to measure an IMT. Cardiorespiratory fitness was measured as VO2max assessed by direct gas analysis.
